# Primary Tracheobronchial Low-Grade Mucoepidermoid Carcinoma: A Report of a Rare Case

**DOI:** 10.7759/cureus.98436

**Published:** 2025-12-04

**Authors:** Gaurav Sahu, Atharva Barve, Hemant Sharma, Pradip Potdar

**Affiliations:** 1 Department of Respiratory Medicine, MGM Medical College Kamothe, Navi Mumbai, Mahatma Gandhi Mission Institute of Health Sciences, Navi Mumbai, IND

**Keywords:** bronchiectasis, mucoepidermoid carcinoma, rigid bronchoscopy, salivary gland neoplasm, tracheal tumor

## Abstract

Primary tracheobronchial tumors are rare entities, with low-grade mucoepidermoid carcinoma (MEC) representing an uncommon subtype that mimics common airway diseases such as asthma or tuberculosis. Early diagnosis is often delayed due to nonspecific clinical and radiological features. We report the case of a 19-year-old female with exertional dyspnea, recurrent nocturnal gasping, and chronic cough who was previously misdiagnosed and treated as pulmonary tuberculosis. Radiological and bronchoscopic evaluation revealed a tracheobronchial endoluminal mass, and histopathological examination confirmed low-grade MEC of salivary gland origin. The patient underwent successful endotracheal debulking through rigid bronchoscopy. This case underscores the importance of maintaining a high index of suspicion for primary airway neoplasms in young patients with chronic or refractory respiratory symptoms.

## Introduction

Primary tracheobronchial tumors constitute less than 0.2% of all respiratory tract neoplasms, with low-grade mucoepidermoid carcinoma (MEC) representing an exceptionally rare subtype of salivary gland-type tumors arising from submucosal glands of the tracheobronchial tree [1]. Initially described by Smetana et al. in 1952, these tumors exhibit a diverse histological spectrum ranging from indolent low-grade to aggressive high-grade forms [2]. Due to their endoluminal growth pattern and central airway location, patients often present with symptoms that mimic chronic bronchitis, asthma, or pulmonary tuberculosis [3]. Radiological findings on chest radiography or CT are often subtle or nonspecific, while definitive diagnosis requires bronchoscopy with tissue biopsy followed by histopathological and immunohistochemical evaluation. Standard treatment primarily involves complete surgical resection, which remains the modality of choice for low-grade lesions.

Here, we present a rare case of tracheobronchial low-grade MEC in a young female, emphasizing the diagnostic and therapeutic challenges.

## Case presentation

A 19-year-old female presented to our tertiary care centre with a four-year history of progressive exertional dyspnea and recurrent nocturnal gasping episodes. She also reported a cough with minimal expectoration for 15 days, associated with significant weight loss of approximately 10 kg over six months and generalized weakness. There was no history of hemoptysis or fever. Notably, she had previously received empirical anti-tubercular therapy for 15 months without microbiological confirmation due to persistent respiratory symptoms.

On physical examination, the patient appeared cachectic with grade 3 digital clubbing. Oxygen desaturation was noted on exertion. Auscultation revealed a monophonic wheeze with right-sided mid-inspiratory crepitations and inspiratory stridors, suggesting upper airway obstruction.

**Figure 1:**
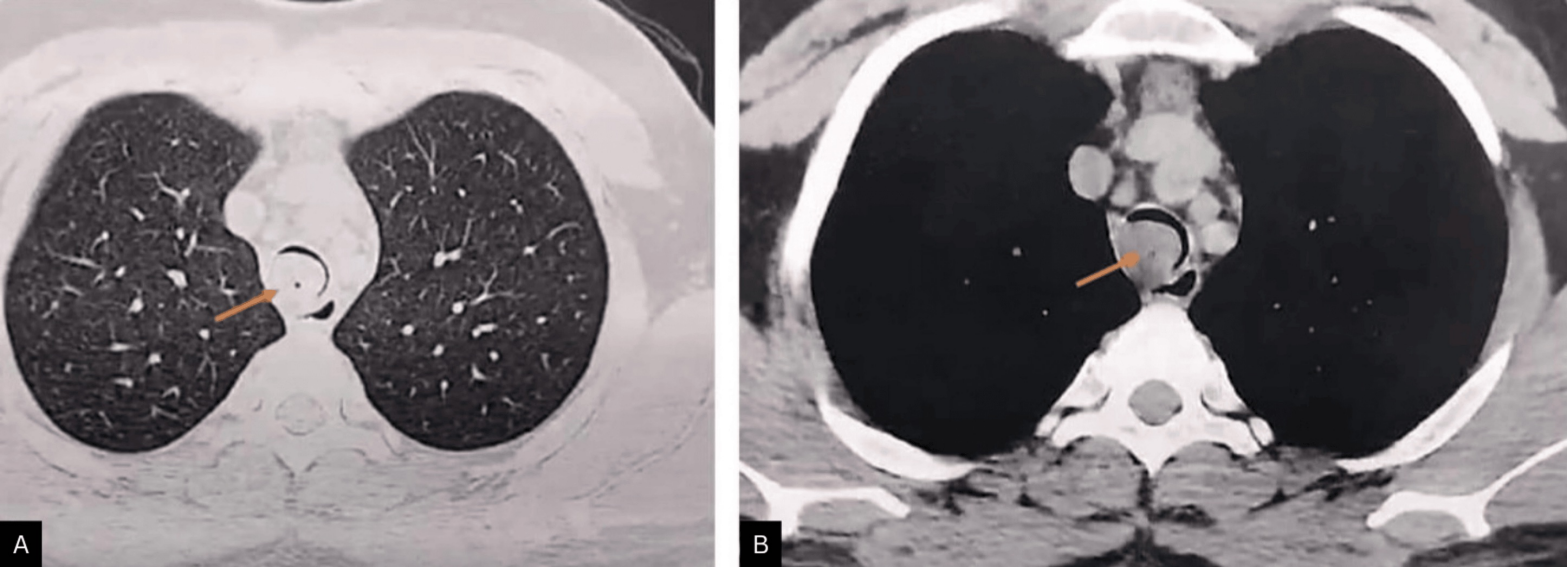
Axial HRCT chest images in lung (A) and mediastinal (B) windows demonstrate a well-defined endoluminal soft-tissue lesion arising from the mid-tracheal wall, causing near-complete luminal obstruction, consistent with a tracheal mass (arrows). HRCT: high-resolution computed tomography

**Figure 2:**
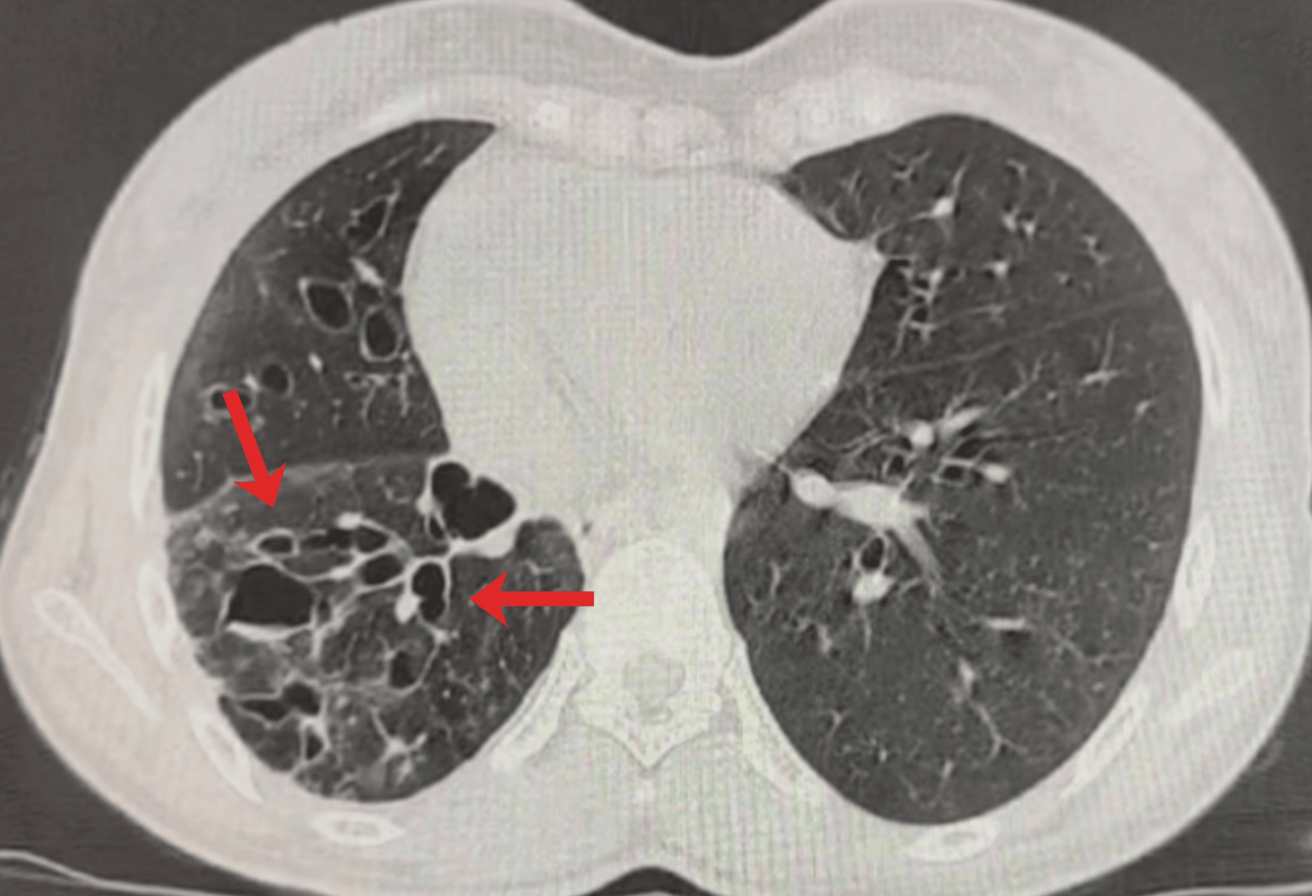
Axial HRCT chest (lung window) demonstrating cystic and varicose bronchiectatic changes (arrows) in the right middle and lower lobes, with mucus plugging and bilateral mosaic attenuation consistent with post-obstructive bronchiectasis secondary to tracheobronchial luminal obstruction. HRCT: high-resolution computed tomography

High-resolution computed tomography (HRCT) of the thorax revealed multiple mucus plugs involving the distal trachea, right main bronchus, and bronchus intermedius. A large endoluminal lesion causing near-complete occlusion of the mid-trachea was visualized (Figure 1). Associated cystic and varicose bronchiectatic changes were noted in the right middle and lower lobes with bilateral mosaic attenuation (Figure 2). Contrast-enhanced computed tomography (CECT) demonstrated a heterogeneously enhancing intraluminal mass extending from the mid-trachea into the right main bronchus.

**Figure 3:**
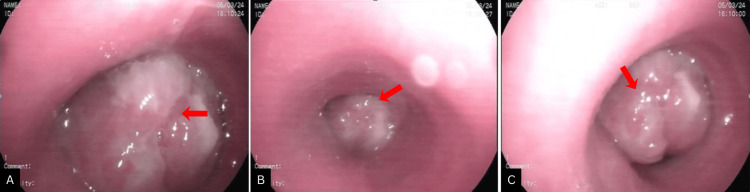
Video bronchoscopy images (A-C) showing a well-circumscribed, reddish-white endoluminal mass arising from the mid-tracheal wall and projecting into the airway lumen (arrows), causing critical narrowing of the tracheal passage. The surface appears smooth and vascular, consistent with a low-grade neoplastic lesion.

**Figure 4:**
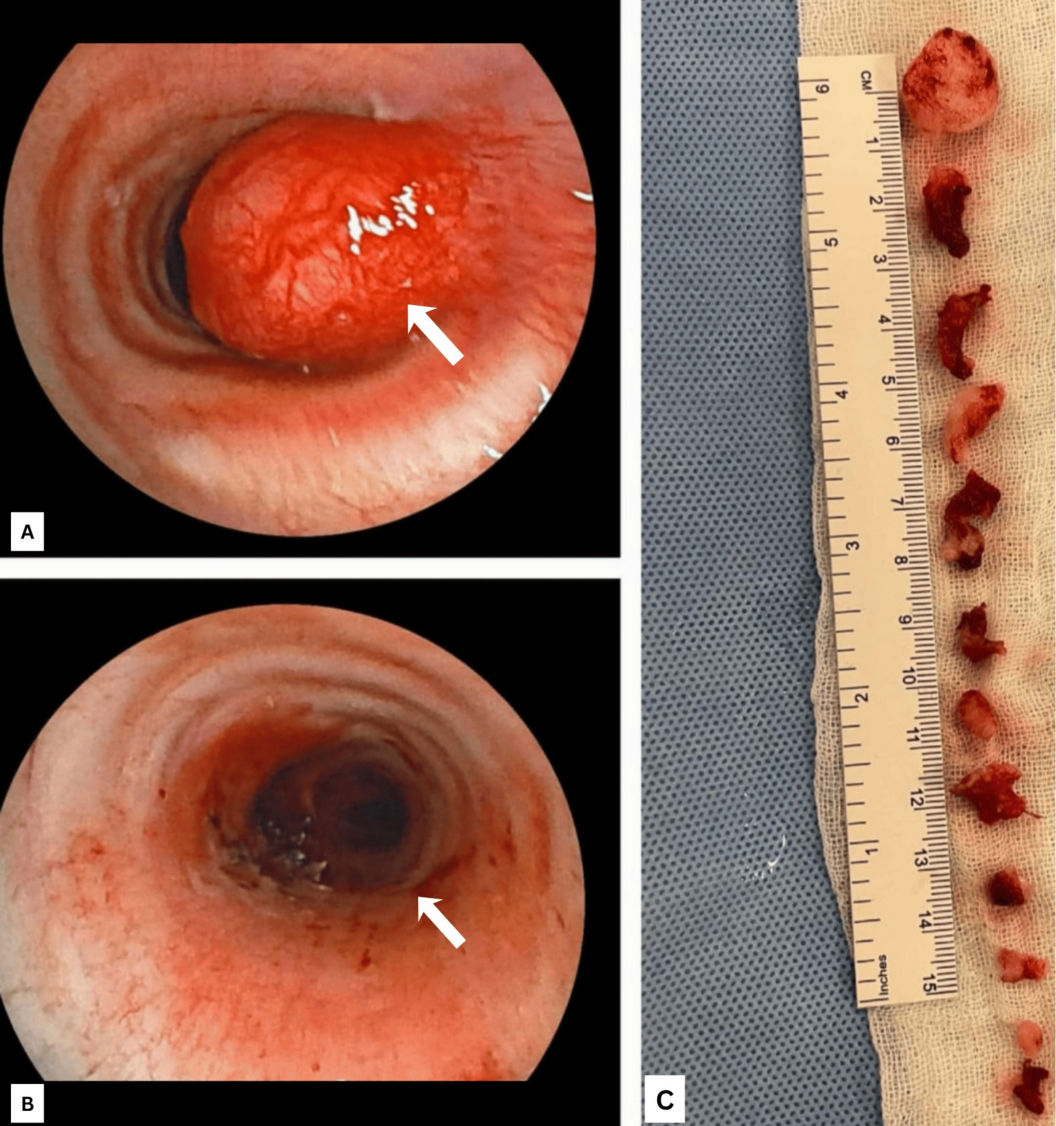
Intraoperative video bronchoscopy images demonstrating tumor debulking performed using a rigid bronchoscope as a conduit. The tracheal mass was excised in a piecemeal manner with a cautery snare, resulting in satisfactory airway patency after complete removal of the obstructing lesion. (A) Bronchoscopic image showing a smooth, reddish-white tracheal mass causing near-complete luminal obstruction (arrow). (B) Post-debulking bronchoscopic view demonstrating a patent airway lumen (arrow). (C) Gross specimen consisting of multiple reddish tissue fragments obtained piecemeal during tumor removal.

**Figure 5:**
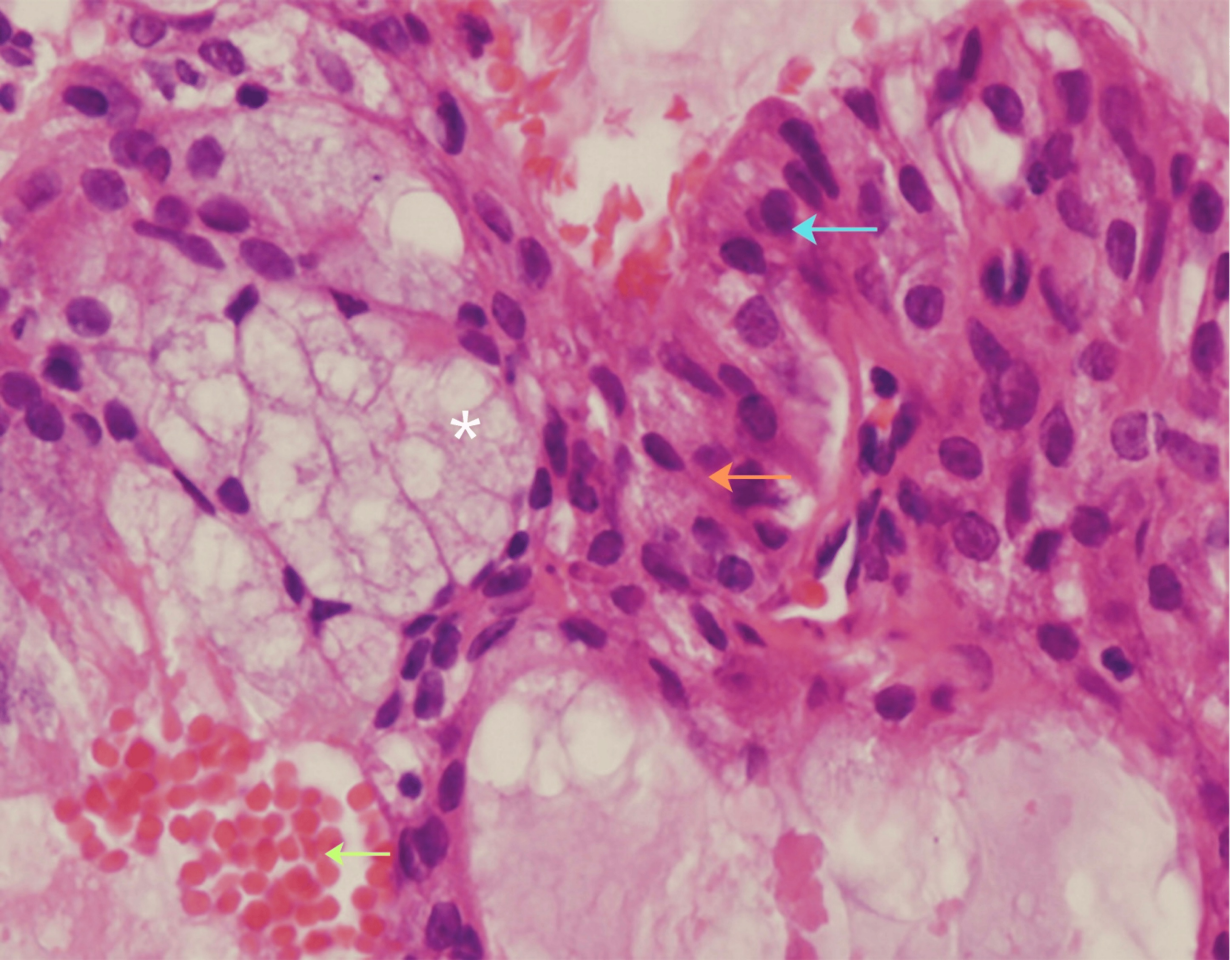
Mucoepidermoid carcinoma (hematoxylin and eosin stain, ×400). The section shows a mixture of mucous cells with vacuolated cytoplasm (*), epidermoid (squamoid) cells with eosinophilic cytoplasm (blue arrow), and intermediate cells (green arrow) with moderately basophilic nuclei. The tumor displays solid nests with cystic spaces containing mucin and scattered erythrocytes (orange arrow). These features are characteristic of a low-grade mucoepidermoid carcinoma.

Video bronchoscopy showed a well-circumscribed reddish-white mass in the mid-trachea causing significant luminal narrowing (Figure 3). Endotracheal mass debulking was performed using rigid bronchoscopy under total intravenous anaesthesia, with piecemeal removal by cautery snare (Figure 4). Histopathological examination revealed epithelial cells arranged in tubules and sheets with large mucin pools and foci of calcification. The adjacent respiratory epithelium showed squamous metaplasia (Figure 5). Mucicarmine stain highlighted abundant extracellular and intracellular mucin. Immunohistochemistry demonstrated positivity for cytokeratin 7 (CK7) and negativity for p40, p63, TTF-1, S-100, and mammaglobin, consistent with a diagnosis of low-grade MEC of salivary gland origin.

The postoperative period was uneventful, and the patient reported marked symptomatic improvement in dyspnea and sleep quality. She remains under regular follow-up with no evidence of recurrence on bronchoscopy or imaging at six months.

## Discussion

Primary tracheobronchial MECs are exceedingly uncommon, accounting for less than 1% of all pulmonary malignancies [4,5]. They arise from submucosal glands of the tracheobronchial tree and are histologically classified into low-grade and high-grade types based on cytological atypia, mitotic activity, and degree of necrosis [6]. Low-grade variants typically display a combination of mucin-producing, squamous, and intermediate cells in a cystic or glandular pattern, often with minimal mitotic activity. Immunohistochemical positivity for CK7 and MUC5AC and negativity for TTF-1 and p63 aid in differentiating MEC from adenosquamous carcinoma and metastatic lesions [7].

Clinically, low-grade MECs frequently present with nonspecific symptoms such as cough, wheeze, dyspnea, and recurrent infections. The prolonged duration of symptoms, as seen in our case, often leads to misdiagnosis as asthma, chronic bronchitis, or tuberculosis - especially in endemic regions [8]. Radiologically, MECs may appear as well-defined endobronchial masses causing obstructive atelectasis or bronchiectasis, but these findings are non-specific. Bronchoscopic evaluation remains the cornerstone for diagnosis and management planning [9].

The mainstay of treatment for tracheobronchial MEC is complete surgical resection with negative margins, which can be achieved by sleeve resection, lobectomy, or bronchoscopic excision depending on tumor location and extent [10]. Low-grade lesions rarely metastasize and generally have an excellent prognosis, with five-year survival rates exceeding 95% after complete excision [11]. In select cases, endoscopic debulking using rigid bronchoscopy can provide significant symptomatic relief and airway patency, particularly when surgical resection is not feasible [12].

Our patient underwent successful bronchoscopic debulking with complete symptom resolution. The absence of recurrence on follow-up highlights the efficacy of this approach in carefully selected low-grade lesions. Nevertheless, long-term surveillance is warranted due to the risk of local recurrence.

## Conclusions

Low-grade MEC of the tracheobronchial tree is a rare but important differential diagnosis for chronic or refractory airway obstruction in young adults. Awareness of this entity is essential to avoid misdiagnosis and delayed treatment. Early imaging, bronchoscopic evaluation, and histopathological confirmation are pivotal for accurate diagnosis. Rigid bronchoscopic debulking offers a safe and effective therapeutic option for localized low-grade lesions with favorable outcomes.
